# Resonant scanning design and control for fast spatial sampling

**DOI:** 10.1038/s41598-021-99373-y

**Published:** 2021-10-08

**Authors:** Zhanghao Sun, Ronald Quan, Olav Solgaard

**Affiliations:** Electrical Engineering, Stanford University, Stanford, CA 94305 USA

**Keywords:** Engineering, Mathematics and computing, Optics and photonics

## Abstract

Two-dimensional, resonant scanners have been utilized in a large variety of imaging modules due to their compact form, low power consumption, large angular range, and high speed. However, resonant scanners have problems with non-optimal and inflexible scanning patterns and inherent phase uncertainty, which limit practical applications. Here we propose methods for optimized design and control of the scanning trajectory of two-dimensional resonant scanners under various physical constraints, including high frame-rate and limited actuation amplitude. First, we propose an analytical design rule for uniform spatial sampling. We demonstrate theoretically and experimentally that by expanding the design space, the proposed designs outperform previous designs in terms of scanning range and fill factor. Second, we show that we can create flexible scanning patterns that allow focusing on user-defined Regions-of-Interest (RoI) by modulation of the scanning parameters. The scanning parameters are found by an optimization algorithm. In simulations, we demonstrate the benefits of these designs with standard metrics and higher-level computer vision tasks (LiDAR odometry and 3D object detection). Finally, we experimentally implement and verify both unmodulated and modulated scanning modes using a two-dimensional, resonant MEMS scanner. Central to the implementations is high bandwidth monitoring of the phase of the angular scans in both dimensions. This task is carried out with a position-sensitive photodetector combined with high-bandwidth electronics, enabling fast spatial sampling at $\sim 100$ Hz frame-rate.

## Introduction

Recent years have seen the rapid development of LiDAR systems in robotics^1^, autonomous vehicles^2–4^ and AR/VR applications^5^. Designing such systems require innovation in both hardware and software because real-time response requires fast information collection and processing. Optical scanners are commonly used in LiDAR to deflect laser beam(s) onto different sampling positions in space and acquire 3D data. Compared to conventional LiDAR scanners that operate in a raster scanning mode, redresonant scanners have a well-known advantage^4,6–8^: the motion amplitude of a resonant scanner is $\sim Q$ times larger than that of a raster scanner, where *Q* is the quality factor of the resonant system^4,6^. Resonant scanning also improve acquisition speed^9–11^. Raster scanners acquire data in a prescribed sequential pattern that is limited by the speed of its slow axis. This results in slow spatial sampling that is unacceptable for many real-world applications, e.g. collision avoidance. In contrast, resonant scanners have high speed in both scanning axes, which is promising for high-speed information collection. To realize this advantage, resonant scanning patterns must be optimized such that information is acquired most efficiently within a short frame time, and their flexibility should be increased to allow situation-dependent, or “random” scanning patterns^12–14^.

Multiple scanning pattern designs have been proposed^9,10,15–18^. Hwang et al. proposed a frequency selection rule for high frame-rate $\sim 10-100$ Hz operation^15,16^. Tuma et al. applied optimization-based scanning trajectory design in scanning probe microscopy^17^, which operates at a lower frame-rate $\sim 1$ Hz. Sub-frame sampling and updating were also proposed to boost the imaging updating rate^6,9,10,19^. These designs tend to focus on actuation frequency selection while ignoring the phase. More recent work discussed both frequency and phase in scanning pattern design^18^, but they are limited to patterns that repeat in each frame. Moreover, none of these designs considered physical constraints such as actuation signal amplitude, which are important in real-world systems. The flexibility in scanning is also critical. As shown in reports on random-access scanning^13,19,20^, scanning patterns that “focus” on specified Regions-of-Interest (RoI) meet data post-processing requirements better than uniform spatial sampling. Resonant scanners cannot abruptly change direction so truly random-access scanning is not possible, and traditionally resonant scanning has been optimized for uniform Field-of-View (FoV) coverage. Therefore, there is a need for approaches that allows RoI focused sampling using resonant scanners.

In this work, we demonstrate optimized designs for resonant scanning patterns with frame-rate $\sim 100$ Hz and limited actuation amplitudes. We first analyze uniform spatial sampling and introduce two metrics: fill-factor and scanning range. We show a trade-off between these two metrics in previous designs to motivate a better solution. An analytical design rule based on unmodulated scanning patterns (both axes have single-tone scanner motion) is proposed that takes various practical considerations into account, such as high frame-rate, bounded actuation amplitude, scanner phase, and pattern repeating period. The proposed design out-performs previous designs that fail to consider these factors. Furthermore, we consider RoI-focused spatial sampling with resonant scanners. For this purpose, we demonstrate the utility of modulated resonant scanning patterns, which contain multiple frequency components around resonance. We develop a task-driven optimization framework to integrate scanning pattern design with post-processing on sampled 3D data.

To demonstrate the applications of designed resonant scanning patterns, we evaluate them in simulated 3D computer vision tasks including LiDAR odometry and object detection^3,21–23^ (section “Simulations”). To experimentally implement the designed patterns, we built a hardware prototype based on a MEMS scanner (section “Experiments”). We developed a control system that stabilizes the scanner phase during operation, which is critical for resonant mechanical systems^8,24–28^. Compared to previously designed high-accuracy, narrow bandwidth phase control systems, the proposed method is wide-band and thus can operates in both unmodulated and modulated scanning modes.

## Scanning Pattern Design

Laser beams reflected from scanners that are resonant in two orthogonal dimensions create “Lissajous patterns” that are described mathematically as follows:

1 $$\begin{aligned} \left\{ \begin{array}{l} x(t) = A_x(t)cos(2\pi f_xt + \phi _x(t))\\ y(t) = A_y(t)cos(2\pi f_yt + \phi _y(t))\\ \end{array} \right. \end{aligned}$$

where $f_x$, $f_y$ are the scanning frequencies, which are assumed to be close to resonant frequencies ($f^r_x$, $f^r_y$). The quantities $A_x(t)$, $A_y(t)$ and $\phi _x(t)$, $\phi _y(t)$ are the amplitudes and phases for the two scanning axes. When amplitudes and phases are static, both *x*(*t*) and *y*(*t*) are single-tone, and we denote the corresponding scanning patterns as unmodulated patterns. When small modulations (or, equivalently, multiple frequency components within resonance bandwidth) are added, we denote the corresponding scanning patterns as modulated patterns. To make the problem of optimizing the scanning patterns tractable, we make the following assumptions: (1) The amplitude of the actuation signal is bounded to reflect limitations on practical hardware. (2) We define a “frame time” $T_{frame}$ (Note that this “frame time” is different from that used in previous literature^9,10^, we provide a comparison between these two concepts in Supplementary Information). Data collected within $T_{frame}$ is used for evaluation or post-processing. We show that the bounded actuation amplitude and short frame time introduce a trade-off between two important metrics for spatial coverage: scanning range and fill factor, and motivates a better design rule. For simplicity, we choose $T_{frame} = m$, *m* being an integer between 6 and 9 (corresponding to $6-9$ ms $T_{frame}$ with a 1 kHz resonant scanner). (3) Without loss of generality, we assume resonant frequencies for the two scanning axes to be $f_x^r = r$, $f_y^r = 1$. In all the simulations, time is scaled by the scanning cycle in the y-axis and is dimensionless. To model typical MEMS scanners, we set a quality factor $Q = 20$. We also limit $r \in [1,3]$. When *r* gets higher, the resonant scanning system gradually transitions to a raster-scanning system, with one axis scanning much slower than the other.

### Unmodulated pattern design

We first analyze the spatial coverage of resonant scanning with bounded actuation amplitude. The goal is to achieve uniform spatial sampling in a normalized $[-1,1]\times [-1,1]$ Field-of-View (FoV). We use fill-factor and scanning range to characterize the spatial coverage of scanning patterns, following common usage in the literature^15,18^. The fill factor characterizes the spatial coverage within a normalized scanning range. The range is determined by the scanning frequencies $f_x$, $f_y$ and the transfer function of the resonant scanner $H_x(f_x)$, $H_y(f_y)$. We quantify the two metrics in Eq. (2):

2 $$\begin{aligned} \left\{ \begin{array}{l} \text {fill-factor} \triangleq 2 - R_{max}, \;\;\;\; \text {higher is better}\\ \text {scanning range} \triangleq H_x(f_x) \times H_y(f_y), \;\;\;\; \text {higher is better}\\ H_x(f_x) = 1 /(\sqrt{((f_x/f_x^r)^2 - 1)^2 + (f_x/(f_x^r Q_x))^2)} Q_x)\\ H_y(f_y) = 1 /(\sqrt{((f_y/f_y^r)^2 - 1)^2 + (f_y/(f_y^r Q_y))^2)} Q_y) \end{array} \right. \end{aligned}$$

**Figure 1:**
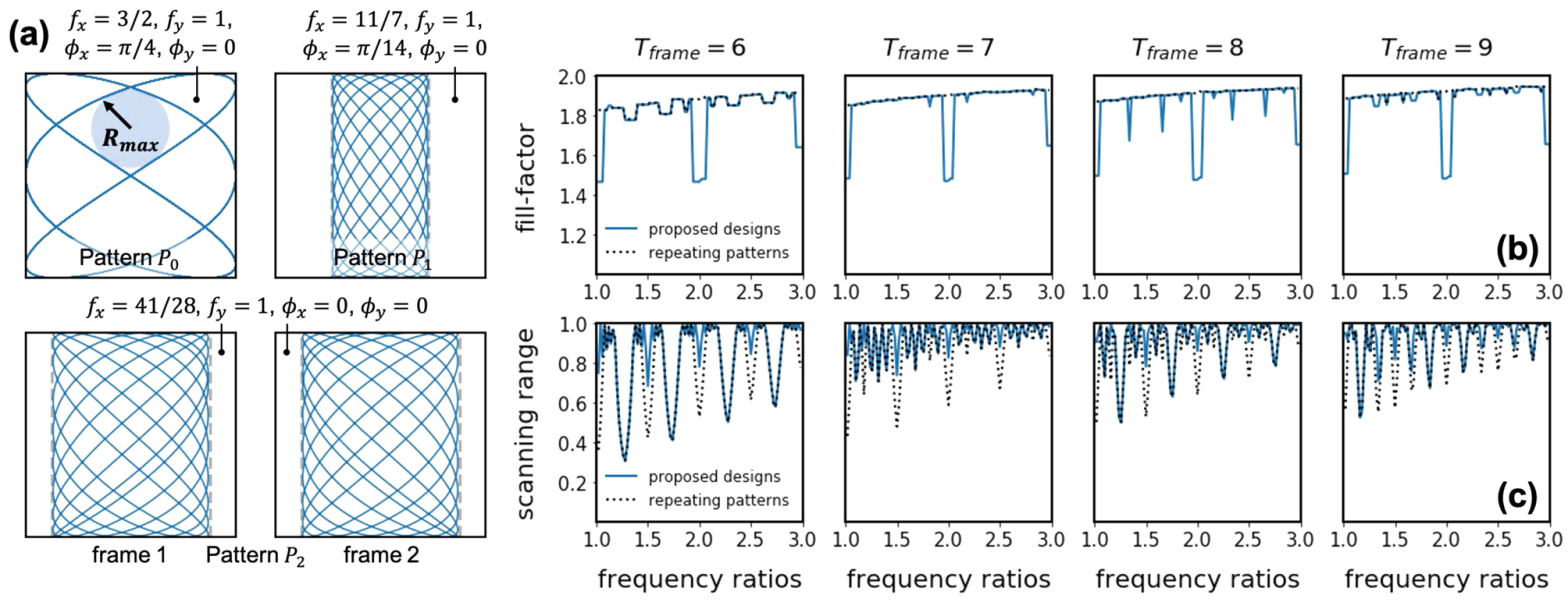
(**a**) Several scanning patterns for a resonant scanner with $f_x^r = 1.5$, $f_y^r = 1.0$ and $T_{frame} = 7$. Pattern $P_0$ is the on-resonance actuated pattern, Although it has large scannning range 1.0, fill-factor is low (0.63), as indicated by the radius of its largest inscribed circle. Pattern $P_1$ uses $f_x = 11/7$, $f_y = 1$, $\phi _x = \pi /14$, $\phi _y = 0$ as parameters. The fill-factor is improved to 0.89 but scanning range is reduced to 0.45. Pattern $P_2$ uses $f_x = 41/28$, $f_y = 1$, $\phi _x = 0$, $\phi _y = 0$, derived from design rule 1. It has fill-factor $=0.88$ and scanning range $= 0.74$. (**b**,**c**) Fill-factor/Scanning range with proposed design rule 1 (blue, solid) and repeating-pattern design rule^18^ (black, dashed), with different resonant frequency ratio and $T_{frame}$ settings.

We normalize the transfer function amplitudes to 1. Note that we only consider the amplitudes of the transfer functions because in the proposed control scheme, we directly monitor phase of the scanner motion, instead of the phase of the actuation signals. Without loss of generality, we assume an ideal harmonic oscillator model for the resonant-scanner because the following analysis is only based on the band-pass characteristic, which are common to all resonant scanners. We also ignore cross-talk between x and y-axis motions in this simplified model. More discussions on cross-talk are presented in the discussion section. Similar to previous literature^17,18^, fill-factor is defined through the radius of the largest inscribed circle $R_{max}$ in the sampling pattern, as shown in Fig. 1a. To decouple the two metrics, the scanning pattern is normalized to $[-1,1]\times [-1,1]$ when calculating $R_{max}$ (See Supplementary Information for details).

With bounded actuation amplitude, there is a fundamental trade-off between fill-factor and scanning range. As an example, we show several scanning patterns with $f_x^r = 1.5$, $f_y^r = 1$, $T_{frame} = 7$ in Fig. 1a. If we actuate on-resonance, the scanning range is at maximum ($=1.0$), as shown in pattern $P_0$. $P_0$ repeats in $t = 2$ with $\phi _x = \pi /4$, $\phi _y = 0$ and it samples on exactly the same trajectory multiple times within $T_{frame}$. This results in a low fill-factor $= 1.63$. On the other hand, if actuated off-resonance, with $f_x = 11/7$, $f_y = 1$, $\phi _x = \pi /14$, $\phi _y = 0$, the sampling pattern has high fill-factor $= 1.89$ (pattern $P_1$). However, off resonance actuation leads to large reduction in scanning range ($= 0.45$). In the lower part of Fig. 1a, we show another design (pattern $P_2$) with $f_x = 41/28$, $f_y = 1$, $\phi _x = \phi _y = 0$. $P_2$ has repeating period $14 = 2T_{frame}$. In each frame, $P_2$ has fill-factor 1.88 and scanning range 0.74. The fill-factor is almost the same as that in $P_1$ and the scanning range ($= 0.74$) is $1.6\times$ larger. Therefore, with respect to spatial coverage within $T_{frame}$, we regard $P_2$ to be a better scanning pattern when compared to $P_1$.

Previous resonant scanning pattern designs generally consider patterns that repeat in each $T_{frame} = m$, with $f_x = k/m$, $f_y = l/m$, $k, l \in {\mathbb {Z}}$^15,18^ and we denote these “repeating patterns”. (Note that the “repeating”/“non-repeating” pattern definition here is different from the definition used in some previous literature^9,10^, we provide a comparison between these two concepts in Supplementary Information) However, for a specific $f_x^r$, $f_y^r$, $T_{frame}$ combination, there might not be a repeating pattern with ($f_x$, $f_y$) close enough to resonance. As in the example in Fig. 1a, it can be verified that $P_1$ is the repeating pattern with ($f_x$, $f_y$) closest to resonance, but $P_1$ still suffers from small scanning range. In this paper, we expand the design space by considering not only repeating patterns, but also patterns with repeating periods longer than $T_{frame}$, such as $P_2$ in the above example. We propose an analytical design rule in design rule 1 to maximize the scanning range while still achieve comparable fill-factors to that of repeating patterns (derivations are provided in Supplementary Information). In the design rule, we search over ($f_x$, $f_y$) pairs around the resonance frequencies (in a close-to-far order) until we find a pair that falls in one of three “good spatial coverage” cases: Case1, where the scanning pattern repeats in $2T_{frame}$ time and a criterion in line 4 of design rule 1 is met. Case2, where the scanning pattern repeats every $T_{frame}$ with $\phi _x = \phi _y = 0$ and Case3, where the scanning pattern repeats in $T_{frame}$ time with $\phi _x \ne \phi _y$. After the frequencies ($f_x$, $f_y$) are chosen, we determine the phases ($\phi _x$, $\phi _y$). The three “good spatial coverage” cases and the criterion in line 4 of design rule 1 guarantee that the scanning trajectory does not repeat within $T_{frame}$. Mathematical proofs for the three “good spatial coverage” cases are provided in Supplementary Information. A very recent paper presented a design rule $|f_x\phi _y - f_y\phi _x|m = \pi /2$ to achieve a high fill-factor for repeating patterns^18^, which is similar to the phase selection rule in Case3. However, design rule 1 is more complete and performs better under the physical constraints.

Figure 1b,c quantitatively show the dependence of fill-factor and scanning range on different settings $r \in [1,3]$ and $T_{frame} \in [6, 9]$. The figures compare the metrics of our proposed designs (blue, solid) and traditional repeating pattern designs^18^ (black, dashed), with a fixed actuation amplitude of 1 in all cases. The comparisons show that: (1) In most cases, the proposed designs have the same fill-factor as the repeating patterns, but larger scanning range. (2) When $T_{frame}$ is shorter, and when *m* has more prime factors, the trade-off is generally less favorable. This is because with $f_x = k/4m$, the greatest common divider (GCD) of *k* and 4*m* are usually larger than 4 and do not fall in the three “good spatial coverage” cases in design rule 1. (3) Integer frequency ratios *r* lead to worse trade-offs between fill-factor and scanning range. More discussions about this special case is provided in the discussion section.

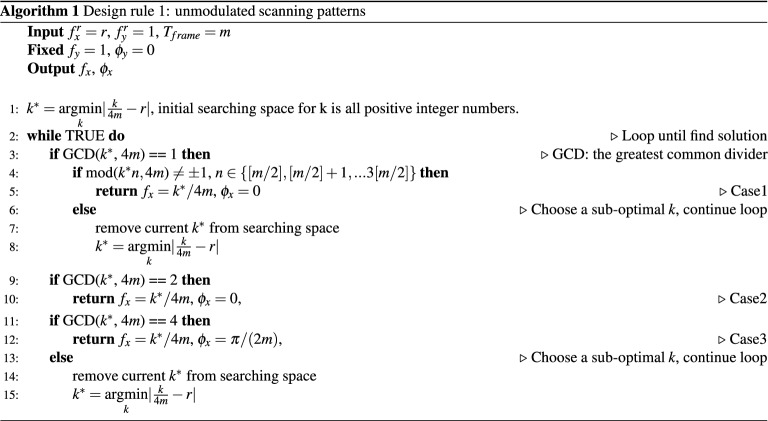



### Modulated pattern design

We further consider a more challenging operation of resonant scanners: Regions-of-Interest (RoI) focusing. In a LiDAR system, through-put of the 3D sensor is fixed, which makes adaptive spatial sampling beneficial for various applications^20,29,30^. More specifically, given a user-defined RoI, we aim at sampling the RoI as densely as possible in all frames. This type of scanning is particularly challenging for resonant scanners and is beyond the capability of the unmodulated scanning patterns, so we propose to use modulated scanning patterns. Such patterns contain multiple frequency components within the resonance bandwidth. However, due to the higher degrees-of-freedom and the complexity of user-defined RoI, analytical design rules are inadequate, so to design modulated scanning patterns, we develop an optimization-based approach. The framework is task-driven because different imaging tasks have different Regions-of-Interest (RoI) for spatial sampling. We seek to improve, by optimized modulation of the parameters, the operation of the resonant scanner as characterized by Eq. (1). However, this model has continuous input parameters so we simplify the model through a Fourier expansion:

3 $$\begin{aligned} \left\{ \begin{array}{l} x(t) = \sum \limits _{n = n_1}^{n_2} \alpha _n H_x(\frac{n}{Lm}) \text {cos}(2\pi \frac{n}{Lm} t) + \gamma _n H_x(\frac{n}{Lm}) \text {sin}(2\pi \frac{n}{Lm} t),\;\;\;\; \sqrt{\sum _n \alpha _n^2 + \gamma _n^2}<= 1\\ {y(t) = \sum \limits _{s = s_1}^{s_2} \beta _s H_y(\frac{s}{Lm}) \text {cos}(2\pi \frac{s}{Lm} t) + \delta _s H_y(\frac{s}{Lm}) \text {sin}(2\pi \frac{s}{Lm} t),\;\;\;\; \sqrt{\sum _s \beta _s^2 + \delta _s^2} <= 1}\\ \end{array} \right. \end{aligned}$$

where $H_x$ and $H_y$ are transfer function amplitudes. We ignore the phases of the transfer functions because they are included in the coefficients of the cosine and sine terms. *m* specifies the frame time $T_{frame}$. $n_1$, $n_2$, $k_1$, $k_2$ defines the number of frequency components in optimization. We find that generally, 5 frequency components give very good optimization results, and in most cases, 3 frequency components are enough. *L* is an integer that controls the spacing of the frequency components. Note that the amplitude constraints in Eq. (3) are equivalent to bounding the root-mean-square ($\text {RMS}$) amplitudes of the actuation signals. This is a looser constraint than bounding the absolute actuation amplitude in unmodulated scanning, which can be expressed as $\sum _n \sqrt{\alpha _n^2 + \gamma _n^2} <= 1$, $\sum _j \sqrt{\beta _s^2 + \delta _s^2} <= 1$.

From Eq. (3), we notice that the scanner motion is linearly determined by the parameter set $\{\alpha _n\}$, $\{\beta _s\}$, $\{\gamma _n\}$, $\{\delta _s\}$. Also, due to the band-pass characteristics of the transfer functions $H_x$, $H_y$, only frequency components close enough to resonant frequencies $f_x^r$, $f_y^r$ have significant impact on scanner motion. This allows efficient optimization of the parameter set. We further discretize time in Eq. (3) to get the sampled scanning pattern $\mathbf{x } \in {\mathbb {R}}^{N}$, $\mathbf{y } \in {\mathbb {R}}^{N}$, where *N* is the number of sampling points. The resonant frequencies $f_x^r, f_y^r$, frame time $T_{frame}$ and *N* are chosen as hyper-parameters in the optimization.

**Figure 2:**
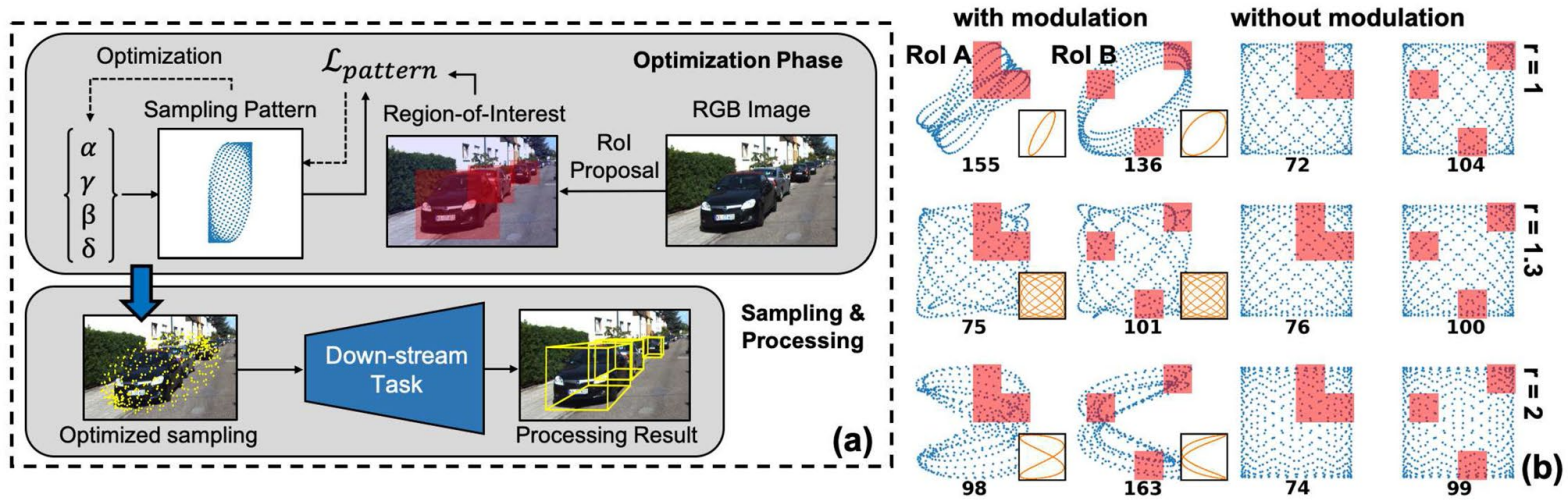
(**a**) Schematic pipeline of the proposed optimization framework. It shapes the sampling pattern into task-specific (or even scene-specific) RoI-focused patterns through objective function ${\mathcal {L}}_{pattern}$. Here 3D object detection is used as a target task. (**b**) Optimization results for different RoI and resonant frequency ratios. Red rectangles show the specified RoI and black numbers under each pattern show the amount of sampling points within the RoI. With $r \sim 1$ and $r \sim 2$, the modulated scanning patterns have denser sampling in RoI, compared to the reference unmodulated scanning patterns. However, with $r \sim 1.3$, this RoI focusing improvement is not significant. At the lower-right corner of each modulated pattern, we also show the corresponding basic unmodulated pattern. (Figure is generated by Microsoft PowerPoint, version 16.49 and Python, version 3.6.8).

The optimization framework is shown in Fig. 2a. First, the parameter set is converted into a sampled scanning pattern $\mathbf{x }$, $\mathbf{y }$. For the specific task (in Fig. 2a, we use 3D object detection as an example), Regions-of-Interest (RoI) are proposed by a fast processing on 2D RGB image, or other heuristic rules and sensing results. The RoI is represented by a weight map $\mathbf{W }$ and its values correspond to the importance of each regions in the FoV. With $\mathbf{x }$, $\mathbf{y }$ and $\mathbf{W }$, we define the objective function ${\mathcal {L}}_{pattern}$ as:

4 $$\begin{aligned} {\mathcal {L}}_{pattern} = \sum _{i,j}^M{\bar{W}}_{i,j}[(x_{i} - \mathbf{x }[n_{i,j}])^2 + (y_{j} - \mathbf{y }[n_{i,j}])^2] \end{aligned}$$

The $[-1,1]\times [-1,1]$ FoV (normalized by the product of amplitudes with on-resonance actuation) is divided into $M\times M$ patches. For each patch (*i*, *j*), we get the closest sampling point ($\mathbf{x }[n_{i,j}]$, $\mathbf{y }[n_{i,j}]$) to its center location ($x_{i}, y_{j}$) and calculate the distance between these two points. ${\bar{W}}_{i,j}$ indicates the importance of each patch and is defined as the average weight in patch (*i*, *j*). Patches with larger average weights have a higher priority during optimization. Note that if the distance between patch (*i*, *j*) and ($\mathbf{x }[n_{i,j}]$, $\mathbf{y }[n_{i,j}]$) is smaller than a threshold, this patch is considered as occupied and ${\bar{W}}_{i,j}$ is set to zero, regardless the weight value in this patch. From ${\mathcal {L}}_{pattern}$, gradient decent optimization^31^ is performed on the parameter set $\{\alpha _n\}$, $\{\beta _s\}$, $\{\gamma _n\}$, $\{\delta _s\}$. Once the optimization is done, spatial sampling can be conducted on a 3D scene, and a sparse point cloud is generated. The sampling is concentrated in the RoI, where most useful information is distributed, and the performance of down-stream tasks is improved. Note that this optimization need not to be done online (e.g. during scanner operation). For some tasks, optimized patterns for different scenes are very similar and thus the optimization process can be done off-line. An example is discussed in Supplementary Information.

Figure 2b shows optimization results for two randomly-selected RoI (RoI A, B) and different resonant frequency ratios. We use $T_{frame} = 7$, total sampling point number $N = 500$ in all optimizations. 5 frequency components are used in the optimization, with $f_x = \{6/7, 13/14, 1, 15/14, 8/7\}f_x^r$, $f_y = \{6/7, 13/14, 1, 15/14, 8/7\}f_y^r$. Similar results are achieved with 3 frequency components $f_x = \{13/14, 1, 15/14\}f_x^r$, $f_y = \{13/14, 1, 15/14\}f_y^r$ and the effect of considering more frequency components (more than 5) within the range of $[6/7, 8/7]f_x^r$, $[6/7, 8/7]f_y^r$ is not significant. The first and the second columns show optimization results with modulated scanning patterns. The third and the fourth columns show reference unmodulated scanning patterns. The modulated scanning patterns have bounded RMS actuation amplitude, as defined in Eq. (3), while the actuation amplitudes for unmodulated patterns are not bounded to better visualize the differences in RoI focused sampling. If the actuation amplitudes of unmodulated patterns are bounded, RoI on the edges and corners can’t be reached in some cases. The comparisons show that: (1) With $r \sim 1$, $r \sim 2$, modulation and optimization lead to an improvement in sampling densities within the RoI, which are shown by the black number under each scanning pattern. (2) The RoI focusing improvement depends on the shape of the specified RoI. With $r \sim 1$, the RoI focusing is more successful for RoI A compared to RoI B, while it is the opposite with $r \sim 2$. (3) With $r \sim 1.3$, the RoI focusing improvement is very limited. The optimization results are only slightly improved compared to the unmodulated scanning patterns.

We provide a qualitative explanation for this dependence of RoI focusing improvement on resonant frequency ratio: A modulated scanning pattern “dithers” around a basic unmodulated scanning pattern, as shown in Fig. 2b at the lower-right corner of each modulated pattern. This basic pattern corresponds to one pair of ($f_x$, $f_y$) in Eq. (3) (it also needs to be in the resonance bandwidth). If the basic pattern has a short repeating period, it only traverse part of the scanning range. For example, with $f_x = 1, f_y = 1$, the scanning trajectory is a simple ellipse. Shape of the basic pattern is controlled by its amplitudes and phases in x and y-axis motion. When appropriate modulations are added, a small shift exists between the scanning trajectories in different repeating periods, and this leads to a focused sampling in the regions close to the basic pattern. However, if the basic pattern has a long repeating period and covers the scanning range uniformly, the modulated scanning patterns can’t be focused onto a certain portion of FoV through optimization. For $r \sim 1$, $r \sim 2$, the repeating period is very short with $f_x = f_y$, $f_x = 2f_y$. However, for $r \sim 1.3$, there does not exist a ($f_x$, $f_y$) pair close enough to resonance while also has a short repeating period (for example, shorter than $t = 2$).

**Figure 3:**
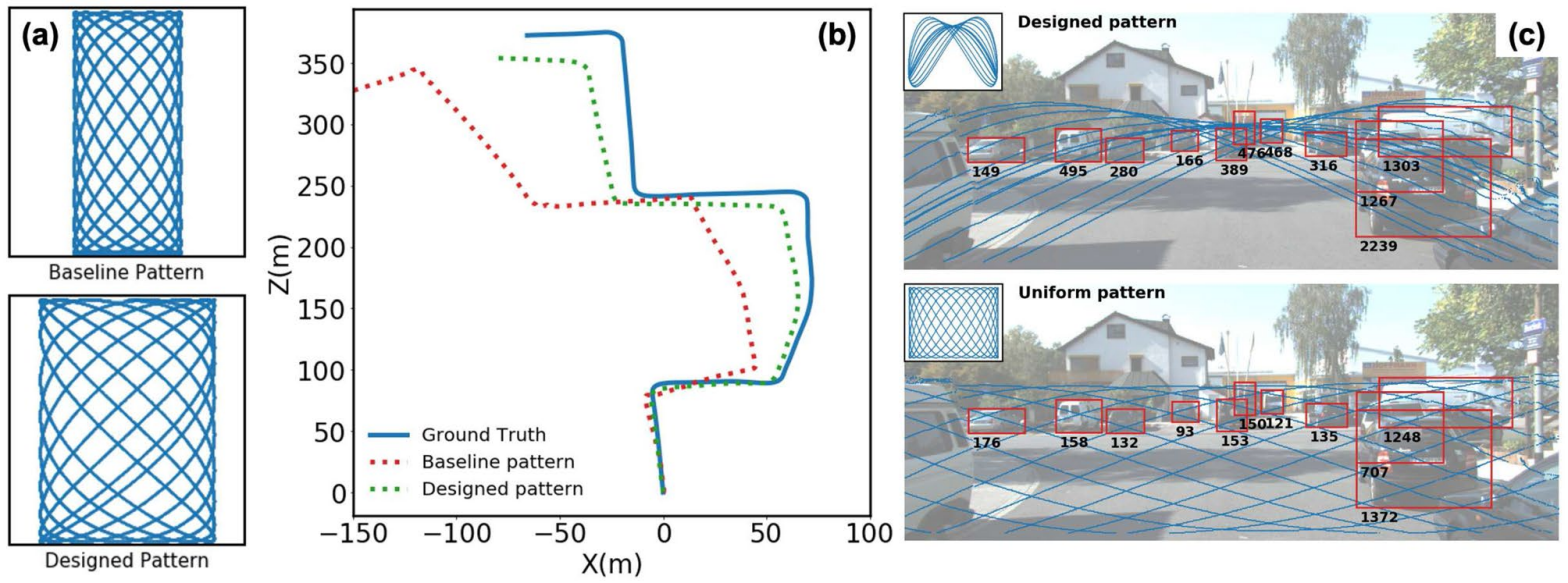
(**a**) Qualitative comparison of baseline and designed scanning patterns ($P_1$ and $P_2$ in Fig. 1a). Scanning range of the designed pattern is $1.6\times$ of that in baseline pattern, leading to a larger perspective field and more reliable feature extraction. (**b**) Trajectory estimations with optimized scanning pattern and baseline scanning pattern. (**c**) Object detection with optimized pattern and baseline pattern. Each red bounding box contains an object and the black number at bottom indicates number of sampling points contained in the bounding box. Due to the RoI-focusing improvement, optimized pattern contains $\sim 3\times$ more sampling points in bounding boxes. The sampling patterns are shown in blue dots.

## Simulations

We evaluate the analytical design rule 1 and the proposed optimization framework in simulated 3D environments^2,32^. Because most 3D imaging datasets currently available are acquired with a raster-scanned or a flash LiDAR, we develop a point cloud generation tool that generates a point cloud corresponding to a resonant scanning pattern. Details of the dataset, implementation and more simulation results are provided in Supplementary Information.

### LiDAR odometry with unmodulated scanning

LiDAR odometry algorithms estimate the trajectory of a moving agent during navigation. They extract feature points from a 3D point cloud acquired in each frame. By comparing the spatial positions of these feature points between successive frames, the position of the agent in a world coordinate can be estimated.

In this work, we consider LiDAR odometry with resonant scanning patterns on the KITTI dataset^2^. We adapt a LiDAR odometry framework, named “LOAM”^3,33^, into the resonant scanning scenario. For comparison, we use the example discussed in Fig. 1a with pattern $P_1$ as the baseline and pattern $P_2$ as the designed pattern. As shown in Fig. 3a, Field-of-View (FoV) of $P_2$ is $\sim 1.6\times$ larger than that of $P_1$. This much larger spatial region gives us more feature points to be observed and processed, which leads to more reliable trajectory estimation (for details of the extracted feature points, please refer to Supplementary Information).

### 3D object detection with moduated scanning

Object detection is another task that is of great interest in 3D computer vision^22,23^. The requirement it imposes on data collection is different from that in odometry. For each scene, important objects (e.g., cars, pedestrians) might concentrate in specific regions in the FoV. Therefore, a denser sampling in these Regions-of-Interest (RoI) is required. As an example, we use hyper-parameters $f^r_x = 1$, $f^r_y = 2$, $T_{frame} = 7$ and number of sampling points $N = 30000$. In Fig. 3c, we show the RoI-focused scanning pattern (upper row). The pattern consists of three frequency components ($f_x = \{\frac{13}{14}, 1, \frac{15}{14}\}$, $f_y = \{\frac{27}{14}, 2, \frac{29}{14}\}$). The relative phases and amplitudes of the three components are optimized to be ($\phi _x = \{86^{\circ }, 178^{\circ }, 86^{\circ }\}$, $\phi _y = \{-96^{\circ }, 145^{\circ }, -96^{\circ }\}$, $A_x = \{0.22, 0.95, 0.22\}$, $A_y = \{0.28, 0.91, 0.28\}$). Using more frequency components in this special case won’t generate significant improvements. When compared to the sampling pattern designed for uniform sampling (lower row), the RoI-focused pattern samples significantly more points ($\sim 3\times$) in regions that contain important objects (cars in this scene). This will largely facilitate the object detection process^23^. We do not conduct quantitative comparisons on object detection, due to the imperfectness in resonant-scanned point cloud generation. However, because of the positive relationship between sampling density and detection accuracy presented in previous literature^2,23^, it is reasonable to expect an increase in accuracy when the optimized scanning pattern is used in real-world LiDAR system.

Note that in this task we do not follow the $f^r_x = r$, $f^r_y = 1$ setting. This is because the dataset we experiment on contains only road scenes. Such a scene is more likely to be symmetric in the horizontal direction compared to the vertical direction. For example, cars are more likely to be on the left and right sides of a road, instead of on the up and down sides of a road. As mentioned above, the performance of RoI focusing depends on the RoI shape. When the axis of symmetry of the RoI shape aligns with that of the scanning pattern, performance is improved. Therefore, we make the scanning pattern also symmetric in horizontal direction by choosing $f^r_x = 1.0$, $f^r_y = 2.0$ instead of $f^r_x = 2.0$, $f^r_y = 1.0$. Optimization results with $f^r_x = 2.0$, $f^r_y = 1.0$ are also presented in Supplementary Information, where the performance is not as good as that in Fig. 3, but still beats the reference unmodulated scanning pattern.

**Figure 4:**
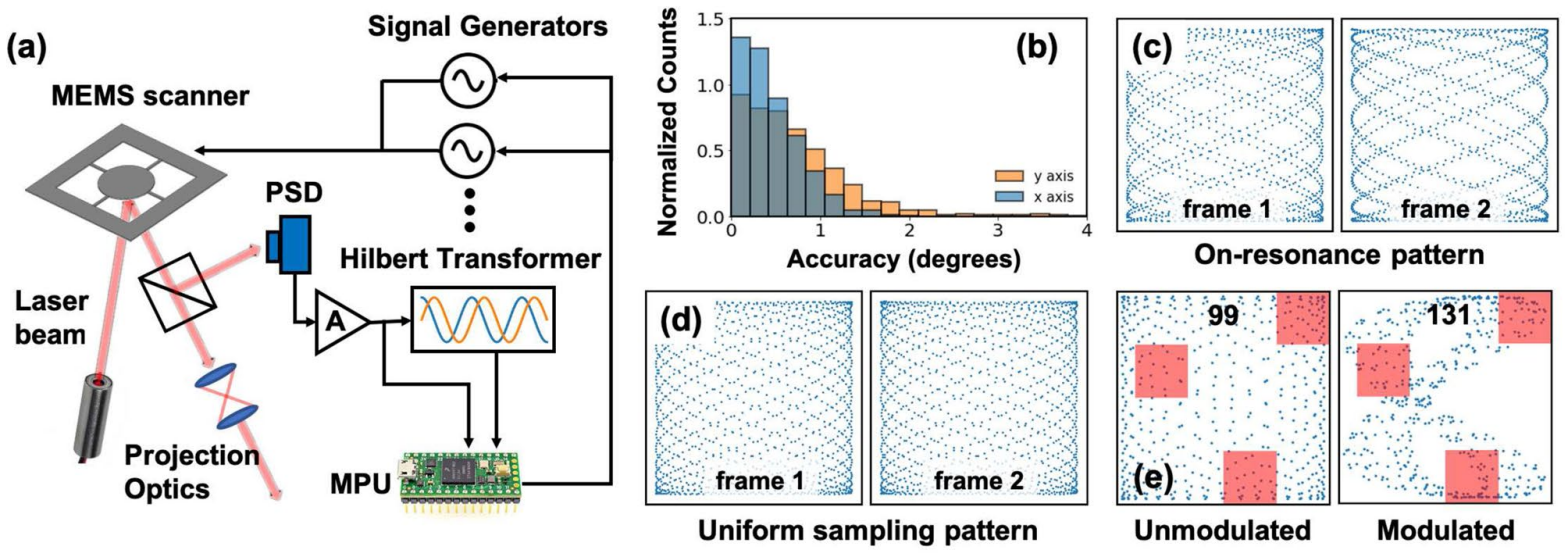
(**a**) Schematic of experimental set up for phase controlled resonant scanning. (**b**) Phase control accuracy of proposed hardware. (**c**) Recorded on-resonance scanning pattern, for two successive frames. (**d**) Recorded designed unmodulated sampling pattern, for two successive frames. (**e**) Recorded modulated sampling pattern with $r \sim 2.0$. Red rectangles are the Regions-of-Interest (RoI) and black numbers indicate amount of sampling points within RoI. Compared to the reference unmodulated scanning pattern, RoI sampling density is increased by $1.3\times$ with modulated scanning.

## Experiments

We implement the designed scanning patterns (Fig. 4a) using a MEMS scanner^34^ with resonant frequencies $f_x^r = 2660$ Hz, $f_y^r = 1100$ Hz, i.e., a resonant frequency ratio $r = 2.42$. The quality factors for the two axis are $Q_x \sim 30$ and $Q_y \sim 50$. Because of the high Q factor and associated low bandwidth, we actuate the y-axis with a single frequency, and restrict modulation to the x-axis. A high-gain amplifier is used to maintain the scanning range when we operate at more than FWHM (Full Width at Half Maximum) away from the resonance. We developed a wide-band phase detection and control system to eliminate the inherent phase uncertainty in MEMS scanners. This uncertainty originates from the environmental sensitivity (e.g. to temperature) of MEMS devices and the strong dependence of the phase on deviations of the resonant frequency^25–28^. With the control system, we achieve $\sim 1^{\circ }$ phase control accuracy, as shown in Fig. 4b. To measure the accuracy, we detect the scanner phase at beginning of each frame and compare it to the required phase, over 10 minutes of scanner operation. This calibration is conducted with a high-speed oscilloscope not shown in Fig. 4a. The accuracy can be improved with faster MPU or better position detection hardware. Phase stability with and without control are further discussed in the Supplementary Information.

### Phase control in unmodulated scanning

We first demonstrate unmodulated scanning. During the experiments, the scanning patterns are recorded with a high-speed position sensor (PSD). We choose $T_{frame} = 6.4ms$, corresponding to $T_{frame} = 7$ in design rule 1. Scanning patterns with on-resonance actuation ($f_x = 2660$ Hz, $f_y = 1100$ Hz) and without phase control are shown in Fig. 4c, for two successive frames. Most portions of the FoV are either over-sampled or under-sampled. Using our proposed design rule, the parameters are changed to $f_x = 2672$ Hz, $f_y = 1100$ Hz, $\phi _x = \pi /14$ and $\phi _y = 0$. The corresponding scanning patterns have much higher fill factor as shown in Fig. 4d.

### Phase control in modulated scanning

To demonstrate modulated scanning, we drive the x-axis at three frequencies $f_x$, $13/14f_x$, $15/14f_x$ and drive the y-axis at a single frequency $f_y$. Phases of the three components in the x-axis scanning are monitored and controlled at the beginning of every 2 frames (when all three phases repeat). Resonant frequencies and frame time are set to be the same as that in the unmodulated scanning experiment. As discussed above, with $r = 2.42$, RoI focusing improvement is limited. Therefore, we go beyond the x-axis resonance bandwidth and select $f_x$ actuation frequency components around 2200 Hz while fix $f_y = 1100$ Hz to emulate a MEMS scanner with $r = 2.0$. We focus the scanning pattern to RoI B in Fig. 2b for demonstration. Due to the high quality factor in y-axis, the degrees-of-freedom in optimization is reduced by $2\times$. However, RoI sampling density in modulated scanning pattern still increases by $1.3\times$ compared to the unmodulated scanning pattern, as shown in Fig. 4e. The experimentally acquired modulated sampling pattern is resampled to 500 sampling points per $T_{frame}$ for comparison with the sampling patterns in Fig. 2.

## Discussion

It is important to note how performance depends on resonant frequency ratios for unmodulated scanning design rule 1 and modulated scanning. Different ($f_x$, $f_y$) pairs generate unmodulated scanning patterns with different repeating periods. For any resonant frequency ratio *r*, pairs of ($f_x$, $f_y$) that correspond to long repeating period always exist in resonance bandwidth^15^. However, a pair of ($f_x$, $f_y$) that corresponds to short repeating period might not exist, as in the case of $r \sim 1.3$. Also, a pair of ($f_x$, $f_y$) that corresponds to repeating period $1/2T_{frame}$, $T_{frame}$ or $2T_{frame}$ do not always exist, as in the case of $r \sim 1$, $r \sim 2$. n the first situation, RoI focusing can’t be achieved while in the second situation, uniform spatial sampling is difficult. In the experiments, we noticed that RoI focusing performs efficiently only with $r \sim 1$ or $r \sim 2$ while these are the worst cases in uniform spatial sampling, as shown in Fig. 1. This result suggests the special usage for resonant scanners with resonance frequency ratio $r \sim 1$, $r \sim 2$ in RoI focused sampling.

Although the proposed scanning pattern designs outperform the baselines, they have the following limitations. First, both designs are based on a moderate quality factor *Q*. If the quality factor is too high, neither the frequency selection rule in design rule 1 nor the modulated scanning pattern designs produce good results. Only small deviations from resonance requires large actuation amplitudes, which is inconsistent with our bounded actuation setting. Second, the optimization problem in modulated scanning pattern design is non-convex. Therefore, our approach does not guarantee convergence to a global optimal. We also assume no cross-talk between x and y-axis scanner motions. This is consistent with the negligible cross talk we observe in our MEMS scanners^34^. If scanners with significant cross talk are employed, then the design rule for unmodulated patterns have to be changed to give good results. ROI focusing, on the other hand, does not need substantial changes to work with scanner that have cross talk. It is straightforward to contain the cross-talk in Eq. (3) and use the optimization framework for both uniform and RoI-focused spatial sampling design.

## Conclusion

Spatial information acquisition is at the heart of many recent advances in the imaging and display industry. A fast and flexible spatial sampling solution will largely improve the robustness and consumer experience. In this paper, we propose resonant scanning pattern design and control schemes that improve the coverage, flexibility, and accuracy in fast spatial sampling. We propose an analytical design rule for uniform spatial sampling, and an optimization-based framework for flexible, Regions-of-Interest (RoI) focused spatial sampling. We also demonstrate the designed scanning patterns in an experimental prototype that applies wide-band control on scanner motion. The proposed methods enable resonant-scanner LiDAR with a high frame-rate $\sim 100$ Hz. When integrated with high-speed point cloud processing algorithms, such systems can be utilized in applications across disciplines, including navigation, robotics, and augmented reality.

## Methods

### Phase control experimental setup

As shown in Fig. 4a, the MEMS scanner is actuated with signal generators (SIGLENT SDG2000X) controlled by external phase modulation signals. The motion of MEMS is detected with a high-speed position sensor (ON-TRAK OT-301). This motion signal is fed into an analog wide-band Hilbert transformer board for 90 degrees phase shift. Both motion signals *x*(*t*), *y*(*t*) and the 90degrees phase shifted signals ${\bar{x}}(t)$, ${\bar{y}}(t)$ are sampled with an MPU chip (PJRC Teensy3.6). In practice, the Hilbert transformer applies a frequency dependent phase shift on both output signals while the relative phase between these two outputs is fixed to be $\pi /2$. We conduct calibrations to remove the phase offset and will ignore it in the following sections. For more details, please refer to Supplementary Information. A fast processing algorithm is performed on the two signals to get the phase and a feed-back signal is generated to the external modulation port of signal generators. Calibrations for each components used in experiment are provided in Supplementary Information.

### Phase calculation process

Phase calculations are simple for the unmodulated actuation case. After collecting *x*(*t*) and ${\bar{x}}(t)$ at the beginning of each frame, a fast arctangent calculation^35^ is performed to get the phase. The detection process takes $\sim 15$ us.

For modulated actuation, phase detection and control is more complicated, because *x*(*t*), *y*(*t*) and ${\bar{x}}(t)$, ${\bar{y}}(t)$ contain multiple frequency components. In this paper, we constrain ourselves to a comparatively simple situation: *x* axis actuation contains three frequency components and *y* axis contains only single frequency component. Similar method can be extended to a more general case. We express the scanner motion in the x-axis as:

5 $$\begin{aligned} \left\{ \begin{array}{l} x(t) = \alpha _0 cos(\omega _x^0t + \phi _x^0) + \alpha _1 cos(\omega _x^1t + \phi _x^1) + \alpha _2 cos(\omega _x^2t + \phi _x^2)\\ {\bar{x}}(t) = \alpha _0 sin(\omega _x^0t + \phi _x^0) + \alpha _1 sin(\omega _x^1t + \phi _x^1) + \alpha _2 sin(\omega _x^2t + \phi _x^2) \end{array} \right. \end{aligned}$$

There are three phases $\phi _x^i, i = 0,1,2$. We detect at both beginning of each frame and at the center of each frame to get six equations:

6 $$\begin{aligned} \left\{ \begin{array}{l} x(0) = \alpha _0 cos(\phi _x^0) + \alpha _1 cos(\phi _x^1) + \alpha _2 cos(\phi _x^2), \\ x(T_{frame}/2) = \alpha _0 cos(\omega _0T_{frame}/2 + \phi _x^0) + \alpha _1 cos(\omega _1T_{frame}/2 + \phi _x^1) + \alpha _2 cos(\omega _2T_{frame}/2 + \phi _x^2), \\ x(T_{frame}) = \alpha _0 cos(\omega _0T_{frame} + \phi _x^0) + \alpha _1 cos(\omega _1T_{frame} + \phi _x^1) + \alpha _2 cos(\omega _2T_{frame} + \phi _x^2), \\ {\bar{x}}(0) = \alpha _0 sin(\phi _x^0) + \alpha _1 sin(\phi _x^1) + \alpha _2 sin(\phi _x^2), \\ {\bar{x}}(T_{frame}/2) = \alpha _0 sin(\omega _0T_{frame}/2 + \phi _x^0) + \alpha _1 sin(\omega _1T_{frame}/2 + \phi _x^1) + \alpha _2 sin(\omega _2T_{frame}/2 + \phi _x^2), \\ {\bar{x}}(T_{frame}) = \alpha _0 sin(\omega _0T_{frame} + \phi _x^0) + \alpha _1 sin(\omega _1T_{frame} + \phi _x^1) + \alpha _2 sin(\omega _2T_{frame} + \phi _x^2), \\ \end{array} \right. \end{aligned}$$

Equation (6) is linear in $\{ \alpha _i cos(\phi _x^i), \alpha _i sin(\phi _x^i) \}, i = 1,2,3$, a fast matrix multiplication is used to solve them. Then we apply the fast arctangent calculation on each (*cos*, *sin*) pair separately to get the phases. The whole data acquisition and processing takes $\sim 40$ us for three frequency components.

## Supplementary Information

Supplementary Information 1.

Supplementary Information 2.

Supplementary Information 3.

## Data Availability

All data used in plotting the figures (including figures in Supplementary Information) are available at https://drive.google.com/drive/folders/1I5_auWKR-UVEHSugAnWAarbaphlW4iKL?usp=sharing.
