# Supplementary material for: Resonant scanning design and control for fast spatial sampling
Source: Sci Rep. 2021 Oct 8;11:20011. doi: 10.1038/s41598-021-99373-y (PMC8501132; doi:10.1038/s41598-021-99373-y)
Supplement: Supplementary file 3 — Supplementary Information 3. [file 41598_2021_99373_MOESM3_ESM.pdf]

Resonant Scanning Design and Control for Fast Spatial Sampling: Supplementary Information

Zhanghao Sun[1,*], Ronald Quan[1], and Olav Solgaard[1]

[1]Stanford University, Electrical Engineering, Stanford, CA, 94305, US
[*]zhsun@stanford.edu

Unmodulated scanning pattern design

Concepts of "repeating pattern" and "frame time"

We note that the terms "repeating pattern" and "frame time" are used in previous literature[1,2] while their definitions are different from those used in this paper. To avoid confusion, here we give a detailed comparison between the concepts:

In previous literature[1,2], a "non-repeating pattern" is defined as a resonant scanning pattern with irrational scanning frequency ratio and never repeats. Any scanning pattern that repeats in limited time is denoted as a "repeating pattern". Accordingly, the "frame time" is defined as the repeating period of a resonant scanning pattern.

In this paper,we focus on optimization of scanning pattern in a short, given time T_{frame}. We denote T_{frame} as "frame time", while it does not necessarily equal the repeating period of scanning pattern. The "frame" here refers to a time period in which one block of data is collected. This data block is processed individually and is not combined with data from other "frames". The "frame time" is a manually given input in the proposed design rule. Practically, it can be the safety response time of an autonomous vehicle and is specified by a higher-level system requirement. The concurrent research[3] shared similar concepts with us. However, they only considered scanning patterns that repeat in each "frame time" (denoted as "repeating pattern" in this paper) and ignore the physical constraint of maximum actuation amplitude in their design. The proposed design rule expands the design space to include patterns that does not repeat in each "frame time" (denoted as "non-repeating pattern" in this paper).

Derivation of unmodulated scanning pattern design rule

The intuitive starting point of proposed unmodulated scanning pattern design rule is:

*The scanning trajectory can't **repeat (or almost repeat)** itself at **middle** of a frame.*

As long as this criteria is met, the scanning pattern within $T_{frame} = m$ has a good fill-factor. Without loss of generality, we make the same assumption as in the main text (since we focus on unmodulated scanning pattern design in this subsection, we ignore the time-dependence of amplitudes A_x, A_y and phases ϕ_x, ϕ_y):

$$\begin{cases} x(t) = A_x cos(2\pi f_x t + \phi_x), \;\; p_x(t) \equiv 2\pi f_x t + \phi_x \\ y(t) = A_y cos(2\pi f_y t + \phi_y), \;\; p_y(t) \equiv 2\pi f_y t + \phi_y \\ f_x = p/q, \;\; p,q \text{ coprime} \\ f_y = 1, \quad \phi_y = 0 \end{cases} \tag{1}$$

Where f_x, f_y, ϕ_x, ϕ_y are the frequencies and phases for two scanning axis. We define two new variables, $p_x(t)$, $p_y(t)$ as the instance phases, for simplicity. We define a set of t such that $\text{mod}[p_y(t),\pi] = 0$. We denote this set of t as T_{nodes}. With the above assumptions, $T_{nodes} = \{n/2\}, n \in \mathbb{Z}$. When $\text{mod}[p_y(t),\pi] = 0$ and $\text{mod}[p_x(t),\pi] = 0$ both hold, the scanning trajectory begins to repeat itself. If t does not coincide with the beginning or end of a frame, the fill-factor of scanning pattern reduces significantly. Also, with $t \in T_{nodes}$, if $|\text{mod}[p_x(t),\pi]|$ is small, the scanning pattern also follows a trajectory that is close to self-repeating. To provide an optimal scanning pattern, we want $|\text{mod}[p_x(t),\pi]|$ to be as large as possible for $\forall t \in T_{nodes}$, except when t is at the beginning or end of a frame. Starting from this observation, we list several conclusions that leads to the three cases in design rule 1:

- First, repeating period of the scanning pattern (equals to q) must be synchronized with the frames. That is, $q = km, k \in \mathbb{Z}^+$. Otherwise, no matter how phases are chosen, the scanning pattern repeats its trajectory at least within some frames. This does not meet the criteria.
- **With $q = m$, which corresponds to Case3 in design rule 1**, selecting the phase ϕ_x is important. With $t \in T_{nodes} = \{n/2\}, n \in \mathbb{Z}$, $\rightarrow p_x(t) = np\pi/m + \phi_x$. Since p,m coprime, $p_x(t)$ value traverses the set $\{k\pi/m + \phi_x\}, k \in \{0,1,2,...m-$

$1\}$. Therefore, the minimum value of $|\text{mod}[p_x(t),\pi]|$ is either $|\phi_x - \pi/m|$ or ϕ_x. If either one of these two values is small, the pattern is close to self-repeating. To make both values sufficiently large, we choose $\phi_x = \pi/(2m)$, such that $|\phi_x - \pi/m|$ also equals to $\pi/(2m)$.

- $q = 2m$ **corresponds to Case2 in design rule 1**. Similar to the above derivations, with With $t = n/2, n \in \{0,1,2,...,2m-1\}$, the minimum value of $|\text{mod}[p_x(t),\pi]|, t \in T_{nodes}$ is either $|\phi_x - \pi/(2m)|$ or ϕ_x. Therefore, we choose $\phi_x = 0$, such that the minimum value of $|\text{mod}[p_x(t),\pi]|$ is only achieved at $t = 0$, the beginning of a frame.

- $q > 2m$, similar to the above derivations, with $t = n/2, n \in \{0,1,2,...,2m-1\}$, the minimum value of $|\text{mod}[p_x(t),\pi]|, t \in T_{nodes}$ is either $|\phi_x - \pi/q|$ or ϕ_x. No matter how we choose ϕ_x, the minimum value of $|\text{mod}[p_x(t),\pi]|$ won't be larger than π/q. Since $q > 2m$, usually fill-factor of the scanning pattern reduces significantly.

- **Heuristically, we find an exception with** $q = 4m$**, which corresponds to Case1 in design rule 1**. Although the minimum value of $|\text{mod}[p_x(t),\pi]|, t \in T_{nodes}$ equals to $\pi/(4m)$, it only happens once and at any other $t \in T_{nodes}$, $|\text{mod}[p_x(t),\pi]| \geq \pi/(2m)$. If $|\text{mod}[p_x(t),\pi]| = \pi/(4m)$ only happens close to beginning or end of frames, the fill-factor reduction can be ignored, as in the example discussed in main text (Figure 1(a), Pattern 2). Therefore, we add an additional check for Case1:

$$\begin{aligned}&\forall t \in T_{nodes}, |\text{mod}[t,m]| > [m/2], \text{ (These } t \text{ values are not close enough to beginning or end of frames),}\\&|\text{mod}[p_x(t),\pi]| \neq \pi/(4m)\end{aligned} \tag{2}$$

- **We also give an explanation on why** $q = 3m$ **and** $q = 5m$ **are not selected**. Similar to Case3, when $q = 3m$, the minimum value of $|\text{mod}[p_x(t),\pi]|, t \in T_{nodes}$ equals to $\pi/(3m)$. It only happens once and at any other $t \in T_{nodes}$, $|\text{mod}[p_x(t),\pi]| \geq 2\pi/(3m)$. If $|\text{mod}[p_x(t),\pi]| = \pi/(3m)$ only happens close to beginning or end of frames, the fill-factor reduction should be tolerable. However, suppose $t_1 \in T_{nodes}$, $|\text{mod}[p_x(t_1),\pi]| = \pi/(3m)$, we have $t_1 + 3m/2 \in T_{nodes}$, $|\text{mod}[p_x(t_1+3m/2),\pi]| = |\text{mod}[p_x(t_1)+p\pi,\pi]| = \pi/(3m)$. The separation between t_1 and $t_1+3m/2$ is $3/2$ of a frame time m. Therefore, it is impossible for both t_1 and $t_1+3m/2$ to be close to beginning or end of frames, which means there are always some frames with low fill-factor.

  Similar explanation is valid for $q = 5m$.

- With $q \geq 6m$, the fill-factor reduction is worse than the case $q = 4m$. This is because even if the minimum value of $|\text{mod}[p_x(t),\pi]|$ is achieved close to beginning or end of frames, part of the kth minimum values, $k \geq 2$ are also small enough to significantly influence the fill-factor. **Therefore, we do not include the cases with** $q > 4m$ **in design rule 1**.

Fill-factor computation for unmodulated scanning patterns

To quantitatively calculate the fill-factor for a scanning pattern, we first scale a scanning pattern into $[-1,1]\times[-1,1]$ range, because fill-factor should not depends on scanning range (size of scanning pattern). Then we sample 1000 points from this scaled pattern, with equal time interval. We divide the $[-1,1]\times[-1,1]$ range into 128×128 patches. For each patch, we search for a minimum distance from its center to the set of sampling points. Finally, we take the maximum value among these minimum distances as R_{max}, radius of the largest inscribed circle.

Phase error tolerance in unmodulated scanning pattern designs

Small uncertainties in phases are unavoidable in real-world system, even with well-designed controls. Here we analyze the phase tolerance of the proposed unmodulated scanning design rule. In Figure 1, we plot the fill-factor vs. resonant frequency ratio $r \in [1,3]$, with a small phase shift $\delta\phi_x$ added to the designed phase ϕ_x. As can be seen, with $\delta\phi_x$, sampling efficiency significantly reduces, but is still at a reasonable value. Also, we noticed that the degradation in sampling efficiency does not grow with the phase error. This is due to the periodic nature of resonant scanning patterns.

Simulation details

We will provide all scripts used in simulations on publication:
https://github.com/zhsun0357/Resonant-Scanned-LiDAR

Dataset generation

We generate the resonant-scanned point cloud from KITTI dataset[4] and NYUV2 dataset[5] for scanning pattern evaluations. For KITTI dataset, we first generate a dense depth map with depth inpainting algorithm[6]. We interpret **x**, **y** in sampling pattern as the normalized yaw angle ϕ and pitch angle θ. With the calibrated transformation matrix between camera coordinate and

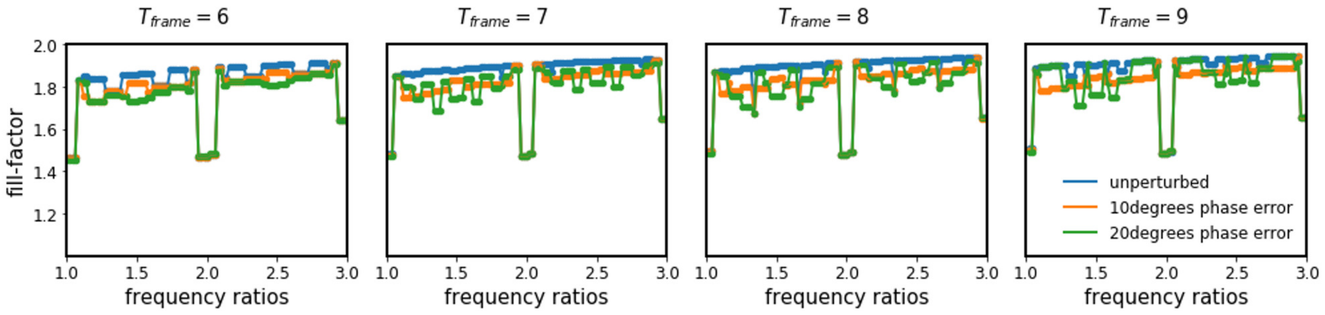

Figure 1. Analysis on phase error tolerance in the unmodulated scanning pattern design. A phase error of 10°/20° are added to ϕ_x when generating the scanning pattern. Compared to the non-perturbed patterns, this phase error results in a degradation in sampling efficiency.

LiDAR coordinate, we are able to sample a 3D point cloud in LiDAR coordinate from the dense depth map with ϕ and θ. There are two major limitations in this point cloud generation process. First, we crop the 360° scanned point cloud in KITTI dataset into an horizontal angle (yaw angle ϕ) range $\sim \pm 40°$, corresponding to the camera FoV. This is because we need the RGB image as reference in depth inpainting[6] while naive depth inpainting without reference RGB image results in large error. Second, due to the pixelization and imperfect estimation in the dense depth map, the resulted resonant-scanned point cloud is geometrically distorted compared to raw point cloud from KITTI dataset. An example of the generated point cloud from KITTI dataset is shown in Figure 2(a), in bird's-eye view. We crop out a local patch in the generated point cloud and compare it to the corresponding reference point cloud from raster-scanned, raw KITTI data. Although the surfaces (e.g., buildings, cars) are generally maintained, small geometric errors can be noticed. This distortion leads to difficulties when implementing object detection on sampled point cloud, because detection results on resonant-scanned point cloud can't be compared with ground truth for raw point cloud. Therefore, we do not show the quantitative comparison on object detection accuracy in the paper. Nevertheless, through the proposed task-driven optimization, enhancement in sampling density in the Regions-of-Interest is evident. Due to the positive relationship between sampling density and detection accuracy presented in previous literature[4,7], it is reasonable to expect an increase in accuracy when the optimized scanning pattern is used in real-world LiDAR system.
For NYUV2 dataset, since a dense depth map is provided, straight-forward sampling on this dense depth map is conducted. Sampling pattern **x** and **y** are directly converted into pixel coordinates in the dense depth map.

Implementation

For LiDAR odometry task, we adapt the framework "LOAM" from previous literature[8]. The trajectory estimation process can be divided into three steps: 1. Divide LiDAR point cloud into multiple scan sections 2. Corner/Surface feature extraction from each section and 3. Trajectory estimation with the extracted features. To better estimate the quality of data collected in a single frame, we do not contain a global mapping step in the algorithm. In Figure 2(b)(c), we show extracted feature points with baseline and designed patterns. Two types of feature points are extracted: corner features that provide in-line geometric constraints and surface features that provide in-plane constraints. A larger Field-of-view (FoV) of designed pattern contains more high-quality feature points and thus makes the odometry estimation more reliable.

Apart from the example of scanning pattern optimization in object detection (shown in main text), we demonstrate the mechanism of the proposed optimization framework with another computer vision task: depth inpainting. Depth inpainting involves generating a dense depth map from a sparsely sampled point cloud (and optionally, a reference RGB image). In this work, we adapt a state-of-the-art depth inpainting framework [9] for task-driven scanning pattern optimization. We use multiple scenes in NYUV2 dataset[5] to get optimized scanning patterns. Regions-of-Interest weight is estimated by the relative absolute error at each pixel during training. Hyper-parameters $f_x^r = 1$, $f_y^r = 1$, $T_{frame} = 7$ and number of sampling points $N = 200$ are used.

As shown in Figure 3(a),(b), it turns out that optimizations with different scenes in the dataset converge to very similar optimal scanning patterns. This is due to the fact that in depth inpainting, each position in the whole FoV is of almost equivalent importance. Therefore, instead of an RoI-focused sampling, a better design for this task might be directly using the unmodulated design. As mentioned in the scanner motion model, in task-driven optimization, the constraint on actuation amplitude is loosened. For the example shown in Figure 3, optimized scanning pattern requires peak actuation amplitude ~ 2.0. To get comparable performance, unmodulated scanning pattern only requires an actuation signal with peak amplitude ~ 1.3.

The depth inpainting framework proposed by Bergman et al.[9] consists of a rough bilateral filter stage[10] and a refinement

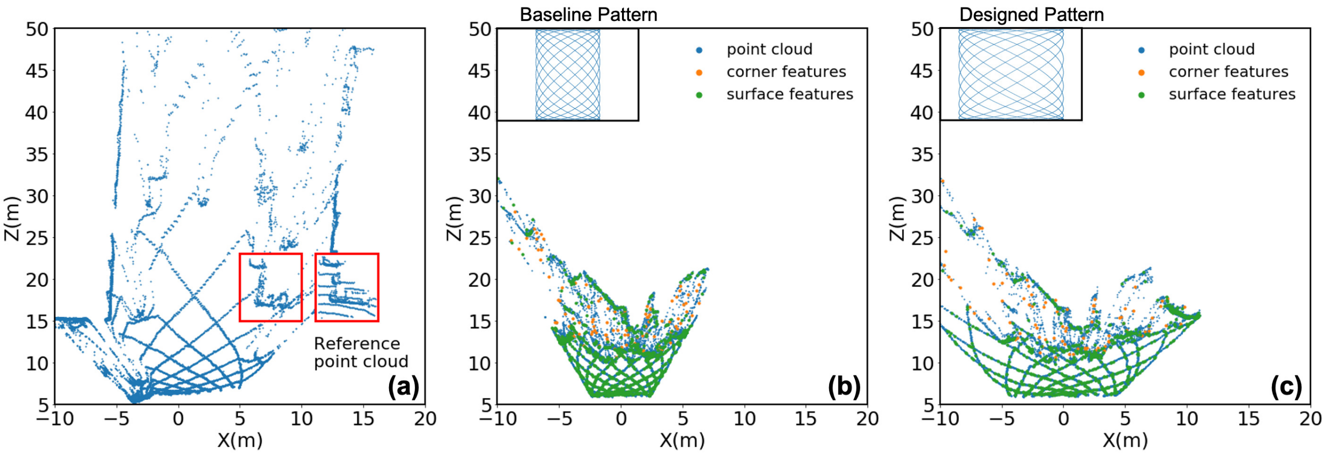

Figure 2. (a) An example generated resonant-scanned point cloud (in bird's-eye view). Note the distortions compared to corresponding raster-scanned "reference point cloud" in KITTI dataset. (b)(c) Feature points extraction (for both surface and corner features) with baseline/optimized sampling patterns in bird's-eye view. Blue points correspond to the full point cloud, orange and green points correspond to corner and surface feature points separately.

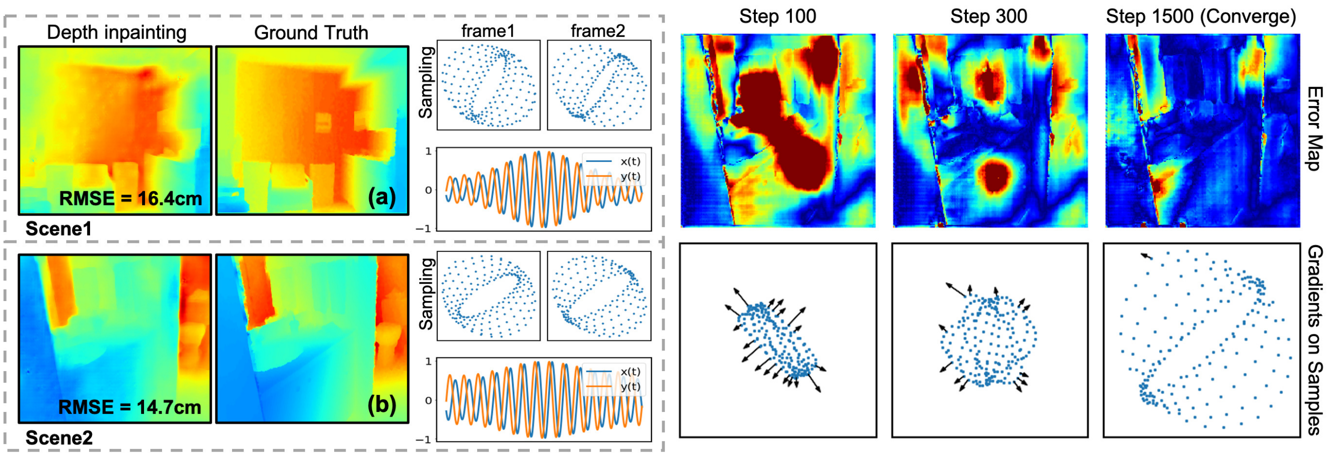

Figure 3. (a), (b) For two scenes in NYUV2 dataset, we show the optimized sampling pattern with $N = 200$ sampling points. We also show the modulated $x(t)$, $y(t)$ with optimized parameter set. On the left part of (a), (b), depth inpainting results with optimal sampling patterns are compared with ground truth depth map. Root-mean-square-errors (RMSE) are listed for each scene. (c) Weight (error) map and gradients on sampling pattern at different optimization steps. Amplitudes and signs of gradients are indicated by the lengths and directions of black arrows. (Figure is generated by Microsoft PowerPoint, version 16.49 and Python, version 3.6.8)

stage. The model also contains a monocular depth estimator[11] to assist the inpainting task. The whole inpainting algorithm is accomplished by an end-to-end convolutional neural network (CNN), where the bilateral filter is also approximated by an CNN model. Instead of that, we use the original bilateral filter in the pipeline, and directly use the error-map from this bilateral filter stage for optimization. The advantage of the proposed optimization framework, compared with that in previous literature[9] is: RoI information across the whole FoV has impact on the scanning pattern updating, even when the non-optimized sampling region is small. Examples of the gradients on sampling pattern at different optimization steps are shown in Figure 3. It can be seen that at each step, gradients on sampling points are "streching" the sampling pattern to uncovered regions. Note that in an early step (step 100), the inpainting result is rough and RoI-weight (error) is even larger in the sampled region. This might be due to the fact that depth reconstruction in these initially sampled regions is coincidentally more difficult. However, occupied patches are set with zero weights in objective function. Therefore, despite this RoI-weight distribution, the optimization

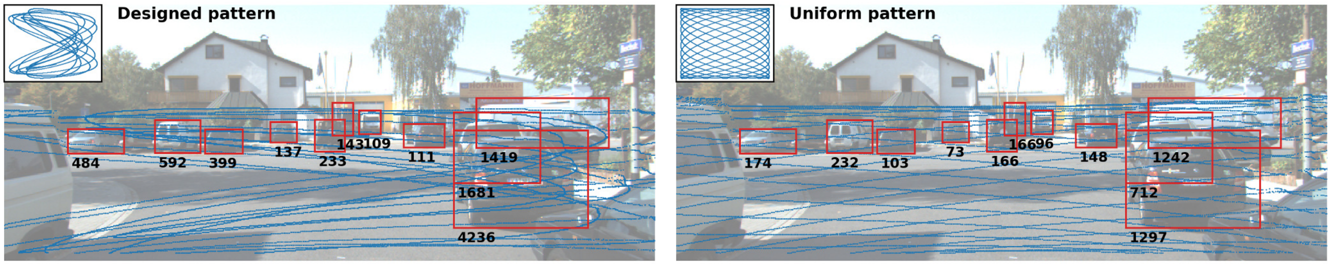

Figure 4. RoI focusing optimization result for 3D object detection with $f_x = 2.0$, $f_y = 1.0$.

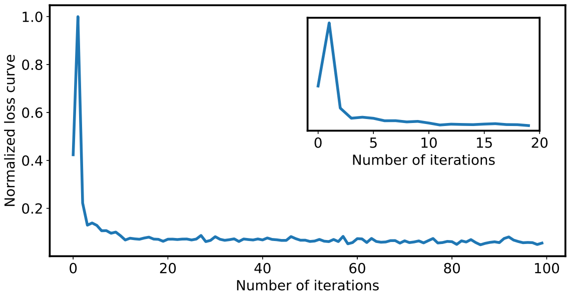

Figure 5. A typical convergence curve in the modulated scanning pattern optimization. The inset is a zoom-in view for iteration steps less than 20. Usually partial convergence is achieved within 10 to 20 iteration steps. With better initialization strategy, the convergence steps can be reduced to < 5 (not shown in the figure)

framework still managed to "expand" the sampling pattern instead of "trapping" it in a local region with large error.

We also show the RoI focusing optimization result for 3D object detection with $f_x = 2.0$, $f_y = 1.0$ in Figure 4. Although the axis of symmetry of the scene does not align with that of the scanning pattern, modulated scanning pattern still out-performs the reference unmodulated scanning pattern significantly.

Considerations in dynamic scenes

In the main text, we focus on optimizing scanning pattern in each data collection period T_{frame} and assume a static environment. In real-world LiDAR applications, adjusting scanning pattern design according to dynamic scenes is also an important functionality. Here we briefly analyze how to adapt the proposed designs to this scenario.

In unmodulated scanning pattern design, the goal is an optimal uniform spatial sampling. With a fixed resonant frequency ratio r and a fixed frame time T_{frame}, the optimal scanning pattern is uniquely given by design rule 1 and is not related to the scene. The designed unmodulated scanning patterns repeat (Case2,3 in design rule 1) or almost repeat (Case1 in design rule 1) in each T_{frame}, regardless the changes in the scene. This is the same case as in most LiDAR systems and we demonstrate its effectiveness in the LiDAR odometry task.

In modulated scanning pattern design, the goal is RoI-focusing and in many cases, the optimized scanning pattern is preferred to change with the scene. When the RoI changes, to make the transition from a previously designed scanning pattern to an updated one, time delay is inevitable. In the proposed scanning pattern design and control framework, this time delay majorly consists of MEMS device response time and scanning pattern design computation time. The MEMS response time is roughly given by $t_{MEMS} = Q/\pi f^r$ under the assumption of ideal harmonic oscillator[12] (Q is the quality factor of the MEMS device, f^r is the MEMS resonant frequency). With $Q = 20$, $f^r = 1$kHz, $t_{MEMS} \sim 6$ms.

Computation time for modulated scanning pattern design depends on multiple factors: complexity of RoI weight map (W in main text, Equation 4), number of iteration steps and the initialization strategy. The weight map can take float values as in the depth completion task (Figure 3), or binary values as in the 3D object detection task discussed in the main text (Figure 3(c)). This choice of RoI weight map parameters depends on the format of input RoI. In 3D object detection task, the RoIs are given as the 2D object bounding boxes estimations. Therefore, binary RoI weight map is sufficient to represent these simple geometric shapes.

With binary RoI weight map, the convergence of optimization framework is much faster and each optimization step size

can be larger, compared to that shown in Figure 3. When started from a random initialization, the algorithm usually takes 10 to 20 iteration steps to achieve convergence. Figure 5(a) shows a typical convergence curve. We implement the optimization algorithm with PyTorch[?] (for automatic gradient descent) and run it on Intel Core i7 CPU (Macbook Pro). Each iteration takes $\sim$ 2.5ms and the total optimization time is $\sim$ 25-50ms. We expect better implementation and more powerful hardware to increase the speed of single optimization iteration.

Although the pattern transition time delay roughly satisfies real-time operation requirement (30 FPS), we can further reduce the time delay for higher speed systems. One of the key insight is that real-world scenes change smoothly. Therefore, a Kalman filter-type prediction algorithm can be utilized to compensate for the time delay. Similar approaches are widely applied in LiDAR data stream processing pipelines[13,14]. Also, since regions-of-interest in successive frames overlap significantly, optimized patterns should also be similar. Therefore, we can use the optimization result in the previous frame as the initialization in current frame optimization. Preliminary experiments show that usually less than 5 iteration steps is needed for convergence, in contrast to $\sim$ 20 iterations when the optimization is randomly initialized. Detailed discussions on these algorithm improvements are out of the scope of the current paper and we leave them for future work.

Experimental details

Calibrations on phase control system

Phase calibration for elements in the control system is required for compensation during the system operation. Calibrations on Hilbert transformer board and position sensor (PSD) are shown in Figure 6. All calibrations are conducted with a high speed oscilloscope. As shown in Figure 6(b), the relative phase between port1 and port2 of Hilbert transformer board is within 90 ± 0.3 degrees range in the operational range of $300 - 3000$Hz.

Phase uncertainty of MEMS scanner

Amplitudes and phases of transfer function $H_x(f_x)$, $H_y(f_y)$ are shown in Figure 7(a),(b), for two scanning axis of the MEMS scanner. From the calibration, resonant frequency of MEMS scanner is determined to be $f_x^r = 2660Hz$, $f_y^r = 1100Hz$. Quality factors are determined from full-width-half-maximum (FWHM) on the transfer function curve, $Q_x \sim 30$, $Q_y \sim 50$. As shown in Figure 7(a),(b), around resonance, relative phase between MEMS scanner motion and the input actuation signal undergoes a steep transition. Small fluctuations in the resonant frequency would result in large phase changes. This small fluctuation can be due to temperature fluctuations, spring stiffening and other random environmental factors.

We characterize this phase uncertainty of MEMS scanner when control system is NOT used. We record the relative phase between the x-axis actuation signal and scanner motion within 40minutes. The MEMS scanner is actuated at a fixed frequency 2660 Hz. No modulation or control are used. Since the phase of actuation signal (from signal generator) is assumed to be stable enough, we attribute the $\sim 10^\circ$ relative phase change, shown in Figure 7(c), to fluctuations in MEMS scanner.

Power consumption

The power consumption of proposed MEMS scanner system can be divided into two parts: control circuit power consumption and MEMS actuation power consumption. Here we give a detailed calculation:
The proposed control circuit consists of 4 operational amplifiers (Op-Amps), 2 for each scanning direction control. This contributes the major power consumption for the control circuit. During operation, each Op-Amp consumes $\sim V_{cc} \times I_{cc}$ power, where V_{cc} is the supply voltage and I_{cc} is the drain current. To achieve a low power consumption, Op-Amp such as TL062 can be used, with drain current as low as 200μA while still operates up to +30 Volts single supply.

The power consumption of a MEMS device can be expressed as $2\pi f C V_{rms}^2$, where f is actuation frequency, C is the capacitor of MEMS and V_{rms} is the RMS(root-mean-square) actuation voltage. Since an electrostatically actuated MEMS device usually has a capacitor $<$ 1pF, and the actuation voltage is in the order of 10-100V, the MEMS device consumes a very small amount of power, in the order of nW to μW. Therefore, to apply such a scanner on portable/mobile devices, the major physical constraint is the maximum amplitude of actuation voltage, instead of the power consumption. When operating off-resonance and maximum actuation voltage is fixed, the scanning range decreases. This physical constraint is ignored in previous designs and motivates the design rule proposed in this paper.

References

1. Newman, J. A. *et al.* Multi-channel beam-scanning imaging at khz frame rates by lissajous trajectory microscopy. In *Three-Dimensional and Multidimensional Microscopy: Image Acquisition and Processing XXII*, vol. 9330, 933009 (International Society for Optics and Photonics, 2015).

2. Sullivan, S. Z. *et al.* High frame-rate multichannel beam-scanning microscopy based on lissajous trajectories. *Opt. express* **22**, 24224–24234 (2014).

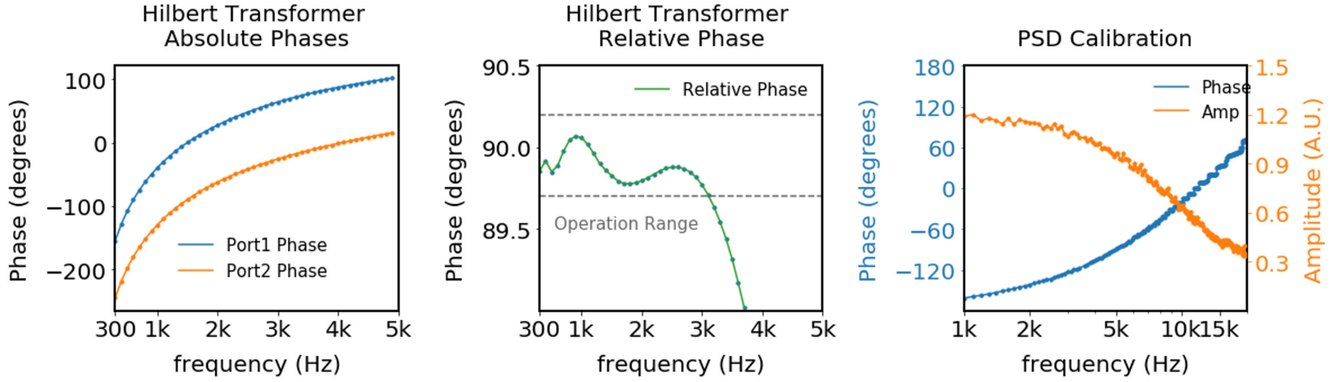

Figure 6. (a) Phase response of port1 and port2 on Hilbert transformer boards. (b) Relative phase between the two ports are within 90 ± 0.3 degrees in the operation range of $300 - 3000$ Hz. This range can be adjusted through changing resistance values in the circuit. (c) Phase and amplitude response of position sensor (PSD).

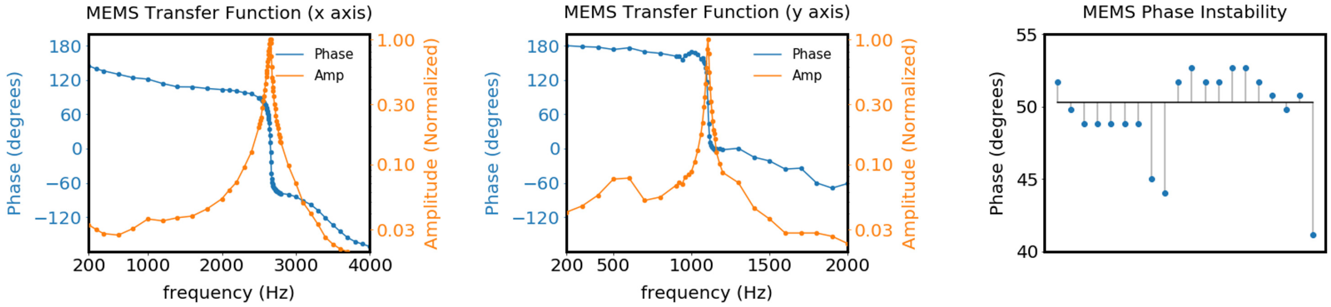

Figure 7. (a),(b) Transfer function amplitudes and phases for the used MEMS scanner. Quality factors $Q_x \sim 30$ and $Q_y \sim 50$ can be estimated from full-width-half-maximum (FWHM) on the H_x, H_y curves. (c) Phase uncertainty of MEMS scanner. Relative phase between MEMS scanner motion and input actuation signal can be unstable due to random fluctuations in the system. An example is shown with 2660 Hz actuation. Relative phase changes can be as large as $\sim 10^\circ$ within 40 minutes time range.

3. Wang, J., Zhang, G. & You, Z. Design rules for dense and rapid lissajous scanning. *Microsystems & Nanoeng.* **6**, 1–7 (2020).

4. Geiger, A., Lenz, P., Stiller, C. & Urtasun, R. Vision meets robotics: The kitti dataset. *The Int. J. Robotics Res.* **32**, 1231–1237 (2013).

5. Silberman, N., Hoiem, D., Kohli, P. & Fergus, R. Indoor segmentation and support inference from rgbd images. In *European conference on computer vision*, 746–760 (Springer, 2012).

6. Ma, F., Cavalheiro, G. V. & Karaman, S. Self-supervised sparse-to-dense: Self-supervised depth completion from lidar and monocular camera. In *2019 International Conference on Robotics and Automation (ICRA)*, 3288–3295 (IEEE, 2019).

7. Qi, C. R., Liu, W., Wu, C., Su, H. & Guibas, L. J. Frustum pointnets for 3d object detection from rgb-d data. In *Proceedings of the IEEE conference on computer vision and pattern recognition*, 918–927 (2018).

8. Zhang, J. & Singh, S. Loam: Lidar odometry and mapping in real-time. In *Robotics: Science and Systems*, vol. 2 (2014).

9. Bergman, A. W., Lindell, D. B. & Wetzstein, G. Deep adaptive lidar: End-to-end optimization of sampling and depth completion at low sampling rates. In *2020 IEEE International Conference on Computational Photography (ICCP)*, 1–11 (IEEE, 2020).

10. Barron, J. T. & Poole, B. The fast bilateral solver. In *European Conference on Computer Vision*, 617–632 (Springer, 2016).

11. Alhashim, I. & Wonka, P. High quality monocular depth estimation via transfer learning. *arXiv preprint arXiv:1812.11941* (2018).

12. https://labcit.ligo.caltech.edu/~ajw/ph106/Files_ph106a/ph106a_2019_L7.pdf.

13. Weng, X. & Kitani, K. A baseline for 3d multi-object tracking. *arXiv preprint arXiv:1907.03961* **1**, 6 (2019).

14. Chiu, H.-k., Prioletti, A., Li, J. & Bohg, J. Probabilistic 3d multi-object tracking for autonomous driving. *arXiv preprint arXiv:2001.05673* (2020).
